# Factors Associated with Quality of Life in Relatives of Adults with Serious Mental Illness: A Systematic Review

**DOI:** 10.1007/s10597-022-00948-4

**Published:** 2022-02-10

**Authors:** Joaquín Salvador Lima-Rodríguez, Alejandro Jesús de Medina-Moragas, María José Fernández-Fernández, Marta Lima-Serrano

**Affiliations:** 1 Department of Nursing. Faculty of Nursing, Physiotherapy and Podiatry, University of Seville, Seville, Spain; 2 Faculty of Nursing, Physiotherapy and Podiatry, University of Seville, Seville, Spain; 3 Andalusian Health Service, Seville, Spain

**Keywords:** Family, Mental disorders, Mental health, Life quality, Serious mental illness

## Abstract

Caring for a family member with a serious mental illness often has an impact on the quality of life (QoL) of caregivers. This could have negative repercussions on their caring skills and thus affect the care provided to that individual. The aim of this paper is to identify current evidence on QoL factors affecting relatives of individuals suffering from serious mental illness. A systematic review related to the research question was conducted in six databases by two independent reviewers. The QoL factors of relatives include sociodemographic, contextual, psychological, physical, and patient factors. The findings are consistent with the results of previous research. Mental health professionals may support a family with a member diagnosed with a serious mental illness by enhancing their education about QoL factors, which would trigger and promote protective factors so that family members could assess and act on them on an ongoing basis.

## Introduction

Serious mental illness (SMI) refers to a heterogeneous group of long-lasting mental disorders that interfere with the ability of the individual to take part in daily life activities and affect daily functioning at work, at home, and in social relationships. SMI is linked to 3 criteria: diagnosis of non-organic psychosis or personality disorder; long duration, defined as a history of two years or more of mental illness or treatment; and disability (Leonhardt et al., 2017). This concept includes schizophrenia, schizoaffective disorder, major depressive disorder, bipolar disorders, and personality disorders (Ministry of Health of the Government of Andalusia, 2020).

The past few decades have been marked by an increase in the deinstitutionalization of patients suffering from SMI, which has led families, by choice or necessity, to assume responsibility for the care of their relatives at home. When an individual is unwell, the relatives often participate in making decisions about the health of the affected individual, thus becoming a potential source of support which, the mental health professional can utilize by actively collaborating in the development of the care process, promoting compliance with the therapeutic plan, and informing the mental health professional about the daily behavior of the patient. In addition, family provides social contact and helps to reduce the risk of relapse (Dewangan et al., 2018; Ropi et al., 2021). However, the participation of family members of patients in caregiving has been identified as something which can create insecurity and ambivalence in the relationship with the patient, changes regarding intimacy and familiarity, shifts in role distribution, lack of opportunity for relaxing activities, sorrow and fears regarding the further course of the illness, impairment of health, and financial strains. This means that the quality of life (QoL) of relatives can also be affected (Bishop & Greeff, 2015; Caqueo-Urízar et al., 2014; Cirici Amell et al., 2018).

Quality of life is defined as an individual’s perception of their position in life in the context of the culture and value systems in which they live and in relation to their goals, expectations, standards, and concerns. QoL involves the assessment of physical health, mental status, autonomy level, social relationships, level of independence, and the external conditions (i.e. the geographical and natural environment, such as money, residence, and information resources) that produce a sense of satisfaction with life (Pinto et al., 2017).

Two widely used tools for assessing QoL are the 36-item Short-Form Health Survey (SF-36) and the World Health Organization Quality of Life assessment (WHOQOL-BREF). The WHOQOL-BREF includes four domains: physical health (pain and discomfort; sleep and rest; energy and fatigue; mobility; activities of daily living; dependence on medicinal substances and medical aids; work capacity), psychological well-being (positive feelings; thinking, learning, memory, and concentration; self-esteem; bodily image and appearance; negative feelings; spirituality/religion/personal beliefs), social relationships (personal relationships; social support; sexual activity), and environment (freedom, physical safety, and security; home environment; financial resources; health and social care: accessibility and quality; opportunities for acquiring new information and skills; participation in and opportunities for recreation/leisure activities; physical environment, i.e. pollution/noise/traffic/climate; transport) (Suárez et al., 2018). In turn, the SF-36 assesses eight health concepts: limitations in physical activities because of health problems; limitations in social activities because of physical or emotional problems; limitations in usual role activities because of physical health problems; bodily pain; general mental health (psychological distress and well-being); limitations in usual role activities because of emotional problems; vitality (energy and fatigue); and general health perceptions (Hagell et al., 2017).

Although the QoL of adults with mental disorders has been extensively studied and proven to be unsatisfactory compared to the general population (Deenik et al., 2017), recent studies have also focused on the QoL of their relatives, revealing that they, too, often experience a worsened QoL (Wong et al., 2012). Research on relatives is therefore important both for relatives themselves and, indirectly, for the health of patients, since a poor QoL would compromise their relatives’ caring skills, thus decreasing the patient’s QoL as well. Considering the increasing demand for family members to give care to relatives with mental illnesses, their QoL and QoL-related factors should be considered (Zendjidjian et al., 2012). We have identified only one review on the QoL of relatives of individuals with a mental illness matching the concept of SMI. That study, performed by Caqueo-Urízar et al. (2009), summarizes the information qualitatively, finding that stress, anxiety, depression, job changes, economic burden, impaired family dynamics, belonging to an ethnic minority, and being a parent to the patient are associated with a poorer QoL in the relatives of individuals with schizophrenia.

The gap found in the literature is the rationale for this review, since no systematic reviews have been identified that include a quantitative assessment of the strength of the association of the different factors with the QoL of this population. For this reason, this paper seeks to address a specific question: “What factors are associated with the QoL of relatives of individuals with SMI?”.

The purpose of the present review was to identify the current evidence on the factors associated with the QoL of relatives of individuals with SMI.

## Methods

### Procedure

Considering the PRISMA standards (Page et al., 2021), a systematic review of the literature was conducted for articles published between 2006 and 2020 to obtain an updated picture of the literature on the topic. The study was registered under number CRD42017062741 in PROSPERO, an international database of reviews in healthcare.

CINAHL, Scopus, Pubmed, PsycINFO, WOS, and ProQuest were the databases searched. The search strategy included the following descriptors: “Mental Disorder,” “severe mental illness,” “Psychosis,” “bipolar,” “depression,” “personality disorder,” “Schizophrenia,” “Family,” “relatives,” “Quality of Life,” and other terms combined with Boolean operators AND and OR.

In order to meet the inclusion criteria, the papers had to be original papers measuring QoL in relatives of individuals with SMI based on quantitative analysis.

All the papers were examined for relevance to the research question and were critically appraised by two independent reviewers (a doctoral student specialized in Mental Health Nursing and a nurse with clinical practice and research experience) who, in case of discrepancy, consulted a third reviewer (a registered nurse with a doctorate degree). In the first screening, the abstracts of the records obtained were read. Then, during the second screening, the complete texts of the papers that had passed the first screening were retrieved and read.

The quality of the resulting papers was appraised using the Basic Research Review Checklist (Rasmussen et al., 2000). The quantitative checklist of this tool contains 24 items grouped into six categories: purpose of study, statement of problem, review of literature, methodology, results and conclusions, and overall concerns.

### Data Analysis

To quantify the relationships between specific factors and the QoL domains of relatives of adults with SMI, standardized statistics were extracted or calculated using the Effect Size Calculator (Wilson, 2001). Cohen’s effect size (*d*), Pearson’s and Spearman’s correlations (*r*), and the coefficient of determination (*R*^*2*^) were used to assist with the interpretation of the strength of the relationship between the different variables and QoL (Hays et al., 2005). Effect sizes are useful because they provide an objective measure of the importance of an effect. The standard interpretations establish that *d* = 0.20, *r* = 0.10, and *R*^*2*^ = 0.01 (accounting for1% of the total variance) indicate a small effect size; *d* = 0.50, *r* = 0.3, and *R*^*2*^ = 0.09 (accounting for 9% of the total variance) indicate a moderate effect size; and *d* = 0.80, *r* = 0.5 and *R*^*2*^ = 0.25 (accounting for 25% of the total variance) indicate a large effect size (Funder & Ozer, 2019). In order to provide relevant results, only results with statistical significance (*p* < 0.05) were included.

All authors of this study certify our responsibility in accepting our conduct of the study and for the analysis and interpretation of the data. We certify our responsibility that we helped write the manuscript and agree with the decisions about it.

## Results

**Fig. 1:**
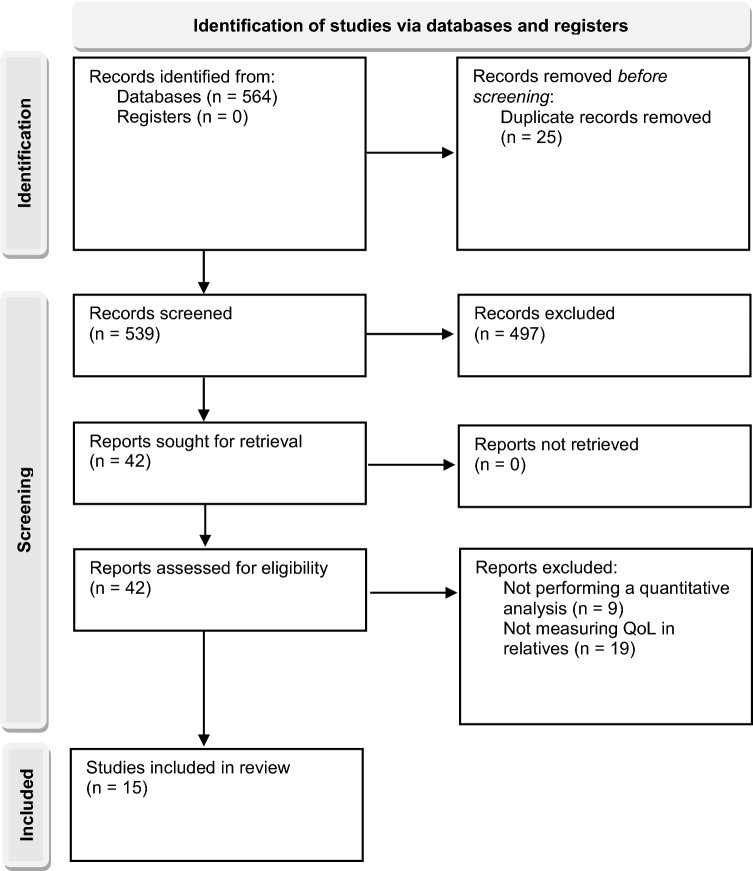
PRISMA flowchart showing the systematic review protocol

Figure 1 is a flowchart showing the number of studies screened, assessed for eligibility and included in the review. Fifteen studies, written in English, met the inclusion criteria. All of them were cross-sectional studies conducted in 14 different countries using self-assessment measures of studies screened, assessed for eligibility, and included in the review. In most of the studies included, these characteristics were analyzed using bivariate analysis (*n* = 12). Eight of the studies used a multivariate analysis, which facilitates a powerful and accurate interpretation of the results.

The samples consisted of relatives of patients with schizophrenia (14), but also with depression (5), bipolar disorder (4), and other mental illnesses (3). The sample sizes ranged from 30 to 286. The mean ages of the family members ranged from 43.7 (*SD* 13.36) to 65.0 (*SD* 7.1). The majority of respondents were parents and female. In order to measure subjective QoL, most studies (7) used the WHOQoL-BREF instrument. The characteristics of the studies included in the review are listed in Table 1. The results of the quality appraisal are shown in Table 2.

**Table 1:** Characteristics of studies included in the systematic review

Author (year) Country	Study characteristics
Angermeyer et al. (2006) Germany	Cross-sectional study; Parents/spouses of patients with Schizophrenia or Depression; *n* = 133; Mean age (SD): 46.9 (12.7); Female gender (%): 44.4%
Boyer et al. (2012) France and Chile	Cross-sectional study; Relatives of patients with Schizophrenia *n* = 286 FRANCE: *n* = 245; Mean age (SD): 60.6 (9.5); Female gender (%): 100%; Parents (%): 67.1% CHILE: *n* = 41; Mean age (SD): 54.3 (15.1); Female gender (%): 100%; Parents (%): 63.4%
Chou et al. (2009) Taiwan	Cross-sectional study; Family carers supporting adults with Mental Illness *n* = 66; Mean age (SD): 65.0 (7.1); Female gender (%): 100%; Parents (%): 89.4%
Gómez‐de‐Regil et al. (2014) Mexico	Cross-sectional study; Relatives of patients with schizophrenia; *n* = 65; Mean age (SD): 48.7 (16.5); Female gender (%): 73.8%; Parents (%): 46.2%; Parnerts (%): 26.2%; Siblings (%): 10.8%; Offspring (%): 9.2%; Other relatives (%): 7.7%
Johansson et al. (2015) Sweden	Cross-sectional study; Parents of an adult child with a history of long-term mental disorder; *n* = 60; Mean age of mothers (SD): 58.8 (9.3); Mean age of fathers (SD): 62 (9.1); Female gender (%): 72%
Kate et al. (2013) India	Cross-sectional study; Relatives of a family member with schizophrenia; *n* = 100; Mean age (SD): 45.9 (11.6); Female gender (%): 45%; Parents (%): 51%; Parnerts (%): 22%; Siblings (%): 20%
Leng et al. (2019) China	Cross-sectional study; Family caregivers of relatives with a SMI; *n* = 96; Mean age (SD): 45.9 (9.2); Female gender (%): 65.8%; Parents (%): 55.2%; Parnerts (%): 23.2%; Offspring (%): 8.9%
Li et al. (2007) China	Cross-sectional study; Family caregivers of relatives with schizophrenia; *n* = 96; Mean age (SD): 47.0 (Not specified); Female gender (%): 57.3%; Parents (%): 53.1%
Lua and Bakar (2011) Malaysia	Cross-sectional study; Family caregivers of relatives with schizophrenia; *n* = 30; Mean age (SD): 51.5 (Not specified); Female gender (%): 46.7%; Parents (%): 53.5%; Parnerts (%): 6.7%; Siblings (%): 13.3%; Offspring (%): 16.7%; Other relatives (%): 10%
Margetic et al*.* (2013) Croatia	Cross-sectional study; Schizophrenia patients’ first degree relatives; *n* = 138; Mean age (SD): 52.6 (14.4); Female gender (%): 63.8%; Parents (%): 76.8%; Siblings (%): 15.2%; Offspring (%): 7.9%
Mizuno et al. (2012) Japan	Cross-sectional study; Family members of patients with schizophrenia; *n* = 34; Mean age (SD): 63.3 (13.3); Female gender (%): 79.4%
Noghani et al. (2016) Iran	Cross-sectional study; Family caregivers of mental disorder patients; *n* = 238; Female gender (%): 72.3%; Parents (%): 41.2%
ZamZam et al. (2011) Malaysia	Cross-sectional study; Family caregivers of relatives with schizophrenia; *n* = 117; Female gender (%): 52.1%; Parents (%): 43.6%
Zauszniewski et al., (2008, 2009) United States	Cross-sectional study; Family members of adults with Severe Mental Illness; *n* = 60; Mean age (SD): 46.28 (11.71); Female gender (%): 100%; Parents (%): 40%; Siblings (%): 23%; Other relatives (%): 37%

**Table 2:** Topics addressed in the basic research review checklist for quantitative studies

	Purpose of study		Statement of problem			Review of literature				Methodology							Results & conclusions					Overall concerns		
	Clarity	Practical Significance	Clarity	Conceptual definitions	Operational definitions	Current citations	Classic studies	Primary sources	Relevance	Population Identified	Adequacy of Sample	Representativeness	Clarity of procedures	Replicability	Data Collection	Data Analysis	Accuracy	Addressed purpose	Significance	Clarity	Limitations	Objectivity	Ethical considerations	Readability
Angermeyer et al. (2006)	+	+	+	−	+	+	+	+	+	+	+	+	+	+	+	+	+	+	+	+	+	+	+	+
Boyer et al. (2012)	+	+	+	−	+	+	+	+	+	+	+	+	+	+	+	+	+	+	+	+	+	+	+	+
Chou et al. (2009)	+	+	+	−	+	+	+	+	+	+	−	+	+	+	+	+	+	+	+	+	+	+	+	+
Gómez‐de‐Regil et al. (2014)	+	+	+	−	+	+	+	+	+	+	−	+	+	+	+	+	+	+	+	+	+	+	+	+
Johansson et al. (2015)	+	+	+	−	+	+	+	+	+	+	−	+	+	+	+	+	+	+	+	+	+	+	+	+
Kate et al. (2013)	+	+	+	−	+	+	+	+	+	+	+	+	+	+	+	+	+	+	+	+	+	+	+	+
Leng et al. (2019)	+	+	+	−	+	+	+	+	+	+	+	+	+	+	+	+	+	+	+	+	+	+	+	+
Li et al. (2007)	+	+	+	−	+	+	+	+	+	+	−	+	+	+	+	+	+	+	+	+	+	+	+	+
Lua and Bakar (2011)	+	+	+	−	+	+	+	+	+	+	−	+	+	+	+	+	+	+	+	+	+	+	−	+
Margetic et al. (2013)	+	+	+	−	+	+	+	+	+	+	+	+	+	+	+	+	+	+	+	+	+	+	+	+
Mizuno et al. (2012)	+	+	+	−	+	+	+	+	+	+	−	+	+	+	+	+	+	+	+	+	+	+	+	+
Noghani et al. (2016)	+	+	+	−	+	+	+	+	+	+	+	+	+	+	+	+	+	+	+	+	+	+	+	+
ZamZam et al. (2011)	+	+	+	−	+	+	+	+	+	+	+	+	+	+	+	+	+	+	+	+	+	+	+	+
Zauszniewski et al. (2008)	+	+	+	−	+	+	+	+	+	+	−	+	+	+	+	+	+	+	+	+	+	+	−	+
Zauszniewski et al. (2009)	+	+	+	−	+	+	+	+	+	+	−	+	+	+	+	+	+	+	+	+	+	+	−	+

*n.s.* not specified, + the topic is addressed, − the topic is not addressed

The different factors related to QoL are grouped into five categories: individual factors, family factors, characteristics of the patient with SMI, factors related to the course of illness, and contextual factors. The associations with QoL have been summarized in Tables 3 and 4 to provide a clearer presentation of the findings. In turn, Table 5 shows the direction of the associations with QoL and the number of papers that explore each factor.

**Table 3:** Factors with significance, effect size and type of analysis in the studies included in the systematic review that used WHOQoL-BREF or QLESQ−SF to assess QoL

Author (year). Measurement	Type of analysis/effect size	Factors with significance					
		Overall QoL	General health	Physical health	Psychological	Social relationship	Environment
Angermeyer et al. (2006) WHOQoL-BREF	Multivariate small to large	n.s	n.s	Age of spouse (*d* = 1.22, *p* = .01), Depression symptoms (*d* = .97, *p* = .01)	Depression symptoms (*d* = .92, *p* = .05), Living situation (*d* = .75, *p* = .042), Sense of coherence (*d* = .76, *p* = .036)	Patient’s functioning (*d* = .87, *p* = .05)	Patient’s diagnosis of schizophrenia (*d* = .44, *p* = .01)
Chou et al. (2009) WHOQoL-BREF	Multivariate small to large	Family support (*β* = .21*, d* = .47, *p* < .05), Stigma (*β* = *− .23, d* = .54, *p* < .05), Physical health (*β* = *− .*39*, d* = .93, *p* < .05)	n.s	n.s	n.s	n.s	n.s
Gómez-de-Regil et al. (2014) WHOQoL-BREF	Bivariate small to large	GAF (*r* = .30, *p* < .01), GHQ-28 (*r* = .74, *p* < 0.01), Somatic symptoms (*r* = .64, *p* < .01), Anxiety-insomnia (*r* = .54, *p* < .01), Social dysfunction (*r* = .58, *p* < .01), Depression (*r* = .52, *p* < .01), Consequences-relative (*r* = .35, *p* < .01), Control-cure of illness (*r* = .28, *p* < .05)	n.s	n.s	n.s	n.s	n.s
	Multivariate small to large	GAF (*β* = − .08, *p* = .015), GHQ-28 (*β* = − .44, *p* < .001); Illness perception (*β* = − .13, *p* = .003), Consequences-patient (*β* = − .0.19, *p* = .041), Consequences-relative (*β* = − .26, *p* = .011), Control-cure of illness (*β* = − .25, *p* = .005), Control-cure by relative (*β* = − .16, *p* = .055)	n.s	n.s	n.s	n.s	n.s
Kate et al. (2013) WHOQoL-BREF	Bivariate small to medium	n.s	Tension (*r* = − .25 *p* = .012)	Tension (*r* = − .22, *p* = .027), Worrying-urging (*r* = .23, *p* = .019), Supervision (*r* = − .23, *p* = .021), Involvement Evaluation (*r* = .33, *p* = .001)	Tension (*r* = − .29, *p* = .002)	Tension (*r* = − .28, *p* = .004)	Tension (*r* = − .29, *p* = .003)
Li et al. (2007) WHOQoL-BREF	Bivariate small to large	Household income (*r* = .28, *p* < .01), Physical health (*r* = .34, *p* < .01), Caregiver burden (*r* = .25, *p* < .05), Demand burden (*r* = .29, *p* < .01), Stress burden (*r* = .37, *p* < .01)	Physical health (*r* = .41, *p* < .01)	Education level (*r* = .23, *p* < .05), Household income (*r* = .28, *p* < .01), Physical health (*r* = .51, *p* < .01), Caregiver burden (*r* = .21, *p* < .05), Demand burden (*r* = .21, *p* < .05), Stress burden (*r* = .35, *p* < .01)	Education level (*r* = .21, *p* < .05), Physical health (*r* = .38, *p* < .01), Caregiver burden (*r* = .38, *p* < .01), Demand burden (*r* = .35, *p* < .01), Stress burden (*r* = .50, *p* < .01)	Physical health (*r* = .24, *p* < .01), Caregiver burden (*r* = .21, *p* < .05), Demand burden (*r* = .21, *p* < .05), Stress burden (*r* = .35, *p* < .01)	Household income (*r* = .36, *p* < .01), Physical health (*r* = .31, *p* < .01), Caregiver burden (*r* = .30, *p* < .01), Demand burden (*r* = .36, *p* < .05), Stress burden (*r* = .39, *p* < .01)
	Multivariate small to large	Physical health (*β* = .385, *p* = .000), Household income (*β* = .24, *p* = .012)	n.s	n.s	n.s	n.s	n.s
Margetic et al*.* (2013) QLESQ − SF	Bivariate small to medium	Age (*r* = − .46, *p* = .0001), Marital status (*d* = .27, *p* = .003), Education level (*d* = .24, *p* = .011), Relationship to the patient (*d* = .56, *p* = .0001)	n.s	n.s	n.s	n.s	n.s
Mizuno et al. (2012) WHOQoL-BREF Medium to large	Bivariate medium to large	Age (*d* = .84, *p* = .08), Sense of coherence (*d* = 1.10, *p* = .011)	n.s	no significant factors	no significant factors	Sense of coherence (*d* = 1.05, *p* = .003)	Age (*d* = .72, *p* = .032), Sense of coherence (*d* = .92, *p* = .02)
	Multivariate medium to large	n.s	n.s	no significant factors	Age (*β* = .42, *p* = .013, Living situation (*β* = − .34, *p* = .042)	no significant factors	Age (*β* = .35, *p* = .032)
ZamZam et al. (2011) WHOQoL-BREF	Bivariate small to large	n.s	n.s	Gender (*d* = .38, *p* = .039), Educational level (*d* = .76, *p* = .001), Employment status (*d* = .56, *p* = .003), Medical problems (*d* = .89, *p* = .000), Relationship to the patient (*d* = .51, *p* = .006), Duration of illness (*d* = .45, *p* = .016), Onset of illness (*d* = .66, *p* = .025), Attending day care (*d* = .65, *p* = .024), BPRS (*r* = .30, *p* = .013)	Patient’s employment (*d* = .62, *p* = .012), Patient’s educational level (*d* = .52, *p* = .008), Caregiver’s educational level (*d* = .57, *p* = .004), Caregiver’s employment (*d* = .38, *p* = .041), Medical problems (*d* = .69, *p* = .001), SRRS (*r* = − .21, *p* = .027), Duration of illness (*d* = .42, *p* = .024), Attending day care (*d* = .63, *p* = .028)	Caregiver’s educational level (*d* = .59, *p* = .006), Patient’s number of hospitalisations (*d* = .73, *p* = .017), Duration of illness (*d* = .68, *p* = .000), Onset of illness (*d* = .66, *p* = .027), Attending day care (*d* = .64, *p* = .025)	Caregiver’s educational level (*d* = .76, *p* = .001), Medical problems (*d* = .47, *p* = .023), SRRS (*r* = − .23, *p* = .012), Patient’s number of hospitalisations (*d* = .74, *p* = .037), Duration of illness (*d* = .43, *p* = .022), Attending day care (*d* = .92, *p* = .003), BPRS (*r* = − .20, *p* = .031)
	Multivariate small to large	n.s	n.s	Caregivers having medical problems (*β* = -.286, *p* = .001), BPRS (*β* = − .204, *p* = .017)	Caregivers having medical problems (*β* = -.197, *p* = .031), Patient’s educational level (*β* = .204, *p* = .026)	Duration of illness (*β* = − .207, *p* = .029), Carer’s educational level (*β* = .203, *p* = .028), Onset of illness (*β* = .195, *p* = .040), Patient’s educational level (*β* = .182, *p* = .046)	Attending day care (*β* = .228, *p* = .009), Carer’s educational level (*β* = .269, *p* = .003), SRRS (*β* = − .182, *p* = .037)

*n.s.* not specified, *GAF* Global Assessment of Functioning, *GHQ-28* general health questionnaire, *BPRS* Brief Psychiatric Rating Scale, *SRRS* Social Readjustment Rating Scale

**Table 4:** Factors with significance, effect size and type of analysis in the studies included in the systematic review that used SF-12 or SF-36 to assess QoL

Author (year). measurement	Type of analysis/effect size	Factors with significance								
		Overall QoL	General health	Physical functioning	Role limitations due to physical health	Bodily pain	Emotional well-being (mental health)	Role limitations due to emotional problems	Social functioning	Vitality (energy/fatigue)
Boyer et al. (2012) SF-36	Bivariate small to medium	n.s	Age (*r* = − .15, *p* < .05), Employment (*d* = .42, *p* < .01), Relationship to the patient (*d* = .30, *p* < .05), Country (*d* = − .29, *p* < .05)	Age (r = − .22, p < .01), Employment (d = .46, p < .01), Relationship to the patient (d = .37, p < .05), Country (d = 0.63, p < .01), Physical composite score: Age (*r* = − .23, *p* < .01), Employment (*d* = .55, *p* < .01), Relationship to the patient (*d* = .44, *p* < .05), Country (*d* = 0.41, *p* < .05)	Age (*r* = − .13, *p* < .05), Employment (*d* = .32, *p* < .05), Relationship to the patient (*d* = .43, *p* < .05), Living situation (*d* = .33, *p* < .05)	Age (*r* = − .15, *p* < .05), Employment status (*d* = .43, *p* < .01), Relationship to the patient (*d* = .36, *p* < .05)	Relationship to the patient (*d* = .45, *p* < .01), Living situation (*d* = .27, *p* < .05), Country (*d* = .35, *p* < .05) Mental composite score: Relationship to the patient (*d* = .44, *p* < .05), Living situation (*d* = .27, *p* < .05)	Relationship to the patient (*d* = .30, *p* < .05)	Relationship to the patient (*d* = .55, *p* < .01)	Relationship to the patient (*d* = .44, *p* < .01), Living situation (*d* = .27, *p* < .05), Country (*d* = .41, *p* < .05)
	Multivariate small to medium	n.s	Relationship to the patient (*β* = *− .*16, *p* < .05)	Age (*β* = − .20, *p* < .05) Relationship to the patient (*β* = *− .*23, *p* < .05), Country (*β* = − .27, *p* < .01) Physical composite score: Relationship to the patient (*β* = *− .*17, *p* < .05), Employment status (*β* = .18, *p* < .05), Country (*β* = − .18, *p* < .05)	Relationship to the patient (*β* = *− .*21, *p* < .05), Living situation (*β* = *− .*29, *p* < .05), Country (*β* = *− .*11, *p* < .05)	Employment status (*β* = *− .*20, *p* < .05), Relationship to the patient (*β* = .15, *p* < .05)	Relationship to the patient (*β* = *− .*23, *p* < .05), Country (*β* = − .13, *p* < .05) Mental composite score: Relationship to the patient (*β* = *− .*22, *p* < .05), Living situation (*β* = *− .*14, *p* < .05)	Relationship to the patient (*β* = *− .*17, *p* < .05), Living situation (*β* = *− .*15, *p* < .05)	Relationship to the patient (*β* = *− .*26, *p* < .01), Country (*β* = − .14, *p* < .01)	Relationship to the patient (*β* = *− .*21, *p* < .01), Country (*β* = − .16, *p* < .05)
Johansson et al. (2015) SF-36	Bivariate small to large	Objective burden (*r* ranged from − .64 to − .14, *p* < .01), Subjective burden (*r* ranged from − .71 to − .21, *p* < .05), Anxiety (*r* ranged from − .86 to − .30, *p* < .01), Depression (*r* ranged from − .82 to − .40, *p* < .01*),* Alienation from provision of professional health care (*r* ranged from − .37 to − .12, *p* < .05)	n.s	n.s	n.s	n.s	n.s	n.s	n.s	n.s
Leng et al. (2019) SF-36	Multivariate large			Care time *(β* = − 15.26, *p* < .05), Financial burden *(β* = − 18.72, *p* < .05), Patient's illness state *(β* = 24.73, *p* < .05), Objective support *(β* = 6.92, *p* < .05)			Patient's marital status *(β* = − 27.09, *p* < .05), Family monthly income *(β* = 13.46, *p* < .05), Patient's illness state *(β* = 24.56, *p* < .05), Coordinating caring, life and work *(β* = − 13.13, *p* < .05), Knowledge about the illness *(β* = 20.94, *p* < .05), Subjective support *(β* = 3.21, *p* < .05) 2.45, Utility of support *(β* = 8.64, *p* < .05)			
Lua and Bakar (2011) SF-36	Bivariate small to large	n.s	Employment (*d* = 1.04, *p* = .011) Health change perceived in the last year: Age (*d* = .88, *p* = .027), Employment status (*d* = .90, *p* = .024)	Age (*d* = 1.08, *p* = .009), Employment (*d* = .92, *p* = .021), Relationship to the patient (*d* = 1.13, *p* = .007), Physical component summary: Age (*d* = .86, *p* = .029), Employment (*d* = .88, *p* = .026), Relationship to the patient (*d* = 1.15, *p* = .006)	Relationship to the patient (*d* = .88, *p* = .027)	Employment (*d* = .78, *p* = .045)	Employment (*d* = .92, *p* = .021), Mental component summary: Employment (*d* = 1.03, *p* = .012)	Employment (*d* = .88, *p* = .027)	Employment (*d* = .88, *p* = .027)	no significant factors
Noghani et al. (2016) SF-36	Bivariate small to medium	Gender (*d* = .42, *p* = .004), Economic status (*d* = .45, *p* < 0.0001), Relationship to the patient (*d* = .20, *p* = .027)	Gender (*d* = .45, *p* = .002)	Gender (*d* = .60, *p* < 0.0001)	no significant factors	Gender (*d* = .32, *p* = .028)	Gender (*d* = .38, *p* = .008)	no significant factors	no significant factors	Gender (*d* = .32, *p* = .028)
Zauszniewski et al. (2008) SF-12	Bivariate small to large	n.s	n.s	Relationship to the patient (*d* = .41, *p* < .05)	no significant factors	no significant factors	Age (*r* = .25, *p* < .05), Patient age (r = .33, *p* < .001); Living situation (*d* = .46, *p* < .05)	no significant factors	no significant factors	no significant factors
Zauszniewski et al. (2009) SF-12	Bivariate Medium to large	n.s	n.s	Depressive cognitions (*r* = − .39, *p* < .001)	no significant factors	no significant factors	Personal resourcefulness (*r* = .53, *p* < .001), Perceived burden (*r* = − .52, *p* < .001), Perceived stigma (*r* = − .36, *p* < .001), Strain (*r* = − .51, *p* < .001), Disruption (*r* = − .52, *p* < .001), Depressive cognitions (*r* = − .69, *p* < .001)	no significant factors	no significant factors	no significant factors

*n.s.* not specified

**Table 5:** Summary of factors associated with QoL in relatives of individuals with SMI

		Direction of association with QoL	Number of papers that include the factor
Individual factors	↑ Age	↑↓	6
	Gender: female	↓	2
	↑ Educational level	↑	3
	Employment status: being employed	↑	3
	Marital status: being single	↓	2
	↑ Personal resourcefulness	↑	1
	↑ Sense of coherence	↑	1
	↑ Physical health	↑	2
	↑ Knowledge about the illness	↑	1
Family factors	Kinship: parent	↓	6
	↑ Family income	↑	3
	Number of dependent members (≥ 2)	↓	1
	Living with the patient	↓	2
Characteristics of the patient with SMI	↑ Age	↑	1
	↑ Educational level	↑	1
	Employment status: being employed	↑	1
	Diagnosis: schizophrenia vs. depression	↑	1
	Better clinical status	↑	2
	↑ Number of hospitalisations	↓	1
	↑ Patient’s functioning	↑	2
Factors related to the disease process	Onset of the illness (≥ 45 years)	↑	1
	Being exposed to the patient’s illness (≥ 10 years)	↓	2
	↑ Illness perception	↓	1
	Perception of illness under their own control	↓	1
	↑ Objective burden	↓	3
	↑ Subjective burden	↓	4
	↑ Psychological distress	↓	1
	↑ Anxiety symptoms	↓	1
	↑ Depression symptoms	↓	4
	↑ Social readjustment	↓	1
	Poor health	↓	1
	↑ Family alienation	↓	1
Contextual factors	Country (Chilean vs. French)	↓	1
	↑ Social stigma	↓	2
	Attending Day care	↓	1
	↑ Social support	↑	2

↑ = positive association with QoL; ↓ = negative association with QoL

### Individual Factors

Some personal characteristics of relatives have been shown to be associated with QoL. These characteristics included age, gender, educational level, employment and marital status, coping, and physical health.

#### Age/Aging

Results related to age are conflicting. Most studies have shown that aging is negatively associated with a poorer physical domain and overall QoL (Angermeyer et al., 2006; Boyer et al., 2012; Lua & Bakar, 2011; Margetic et al., 2013; Mizuno et al., 2012). Conversely, other studies found that aging was positively associated with better psychological and environment domains (Mizuno et al., 2012; Zauszniewski et al., 2008).

#### Gender

Males had better QoL than females in the physical domain, overall QoL, bodily pain, general health, vitality, and mental health (Noghani et al., 2016; ZamZam et al., 2011).

#### Educational Level

Positive associations were found with the physical, psychological, social, and environment domains, which are greater at higher educational levels (Li et al., 2007; Margetic et al., 2013; ZamZam et al., 2011).

#### Employment Status

Being employed was positively associated with a higher QoL (ZamZam et al., 2011). Specifically, this factor had positive associations with general health, physical and psychological domains, bodily pain, role-physical, role limitation-emotional problems, social functioning, mental health, health change perceived in the previous year, and the physical and mental component summaries (Boyer et al., 2012; Lua & Bakar, 2011).

#### Marital Status

Being single, compared to being married, is associated with poorer QoL (Leng et al., 2019; Margetic et al., 2013).

#### Coping

Personal resourcefulness, which includes skills for learning to cope with adverse life experiences and self-help strategies and knowledge used in specific situations, was positively associated with mental health (Leng et al., 2019; Zauszniewski et al., 2009). In addition, Sense of coherence (a pervasive and enduring feeling of comprehensibility, manageability, and meaningfulness) was shown to be associated with the psychological, social relationships, environment, and overall QoL domains (Mizuno et al., 2012).

#### Physical Health

Physical health of relatives was associated with higher QoL in the social relationship, environment, general, physical, and psychological domains of QoL (Li et al., 2007; ZamZam et al., 2011).

### Family Factors

Among the factors inherent in the family are the relationship of kinship with the patient, the economic status of the family, the number of dependent members in the family, and coexistence with the patient.

#### Kinship

According to different authors, being a parent, in contrast to having any other relationship with the individual with a mental disorder, is generally related to a poorer QoL, which has an impact on physical health, role-physical, the physical and mental component summaries, vitality, bodily pain, role-emotional problems, mental health, social functioning, and overall QoL (Boyer et al., 2012; Lua & Bakar, 2011; Margetic et al., 2013; Noghani et al., 2016; ZamZam et al., 2011; Zauszniewski et al., 2008).

#### Family Income

The higher the economic status, the higher the QoL, specifically in the physical and environment domains (Leng et al., 2019; Li et al., 2007; Noghani et al., 2016).

#### Number of Dependent Members in the Family

Having fewer than two dependent members in the family is also related to a better QoL of the relative in the social domain (ZamZam et al., 2011).

#### Living Situation

Compared to living apart, living with the individual with mental disorder has been shown to be related to the relatives’ poorer QoL in the psychological, mental composite score, role-physical, role-emotional problems, and vitality domains (Boyer et al., 2012; Zauszniewski et al., 2008).

### Characteristics of the Patient with SMI

Certain factors related to the individual with SMI, such as age, educational level, employment status, functioning, diagnosis, clinical status, or number of hospital admissions, are associated with the QoL of family members.

#### Sociodemographic Characteristics of the Patient

The aging of patients was reported to be associated with a higher QoL of the family member (Zauszniewski et al., 2008). In addition, the higher educational level of the patient was shown to be positively associated with the psychological and social domains of the relative (ZamZam et al., 2011). Finally, the employment status of the patient was also associated with the QoL of the relative, which promoted the psychological domain.

#### Diagnosis of the Patient

Differences have been found between the diagnoses with respect to QoL. These were associated with a higher environment domain in cases in which schizophrenia, and not depression, was the diagnosis of the adult with SMI (Angermeyer et al., 2006).

#### The Patient’s Clinical Status

This factor was shown to be associated with poorer physical, mental and environment domains (Leng et al., 2019; ZamZam et al., 2011). In addition, the number of hospitalizations was also associated with the physical, psychological, social, and environment domains. The impairment of the patient’s functioning was associated with higher social relationships and QoL of the relative (Angermeyer et al., 2006; Gómez‐de‐Regil et al., 2014).

### Factors Related to the Course of Illness

During the course of illness, in which the family participates, several factors appear: the duration of the course of illness, the perception of the illness on the part of the relatives, the objective and subjective burden of the relatives, psychological distress, and family alienation.

#### Duration of Exposure to the Patient’s Illness

An onset of the illness from the age of 45 or older was associated with higher QoL in the social relationship and physical domains. However, being exposed to the patient’s illness over 10 years or more had negative associations in the social, physical, psychological and environment domains (ZamZam et al., 2011).

#### Illness Perception of the Relatives

A high level of illness perception was related to a poorer QoL. Being aware of the consequences that a mental illness has for the relative and the perception of chronic illness were related to a poorer QoL. Finally, the relative’s perception of illness as being under the control of the patient and/or the treatment, rather than under their own control, showed a higher QoL (Gómez‐de‐Regil et al., 2014).

#### Objective Burden

Problems and changes in family life (household routine, relationships, and leisure time) were associated with a poorer Overall QoL in the physical, psychological, social, and environment domains (Johansson et al., 2015; Li et al., 2007).

#### Subjective Burden

Negative feelings and the mental health status of the relatives, including factors such as subjective demand burden, subjective stress burden, Worrying-urging, Tension, Supervision, Strain, and Disruption, were reported to be associated with a lower overall QoL and its various domains (Johansson et al., 2015; Kate et al., 2013; Li et al., 2007; Zauszniewski et al., 2009).

#### Psychological Distress

Somatic symptoms, anxiety-insomnia, social dysfunction, and depressive symptoms were found to be associated with QoL and social dysfunction domains (Gómez‐de‐Regil et al., 2014). Anxiety was shown to be related to physical functioning, mental and general health, role-emotional, social functioning, vitality, role-physical, and bodily pain (Johansson et al., 2015).

Depression symptoms in relatives of adults with a mental illness were found to be associated with QoL and its domains in different studies (Angermeyer et al., 2006; Gómez‐de‐Regil et al., 2014; Johansson et al., 2015; Zauszniewski et al., 2009). In addition, social readjustment, as an individual’s experience of psychosocial stress, was reported to be associated with the social relationships subscale (ZamZam et al., 2011). Finally, the caregiving relative’s poor health (mobility, self-care, daily activities, pain/discomfort, and anxiety/depression) has also been found to be associated with the relative’s QoL (Chou et al., 2009).

#### Family Alienation

Feeling alienation from the provision of professional healthcare, which may consist of powerlessness and social isolation, was shown to be associated with poorer mental and general health, role-emotional, social functioning, vitality, bodily pain, and role-physical (Johansson et al., 2015).

### Contextual Factors

Country of origin, stigma, and perceived social support are studied as contextual factors associated with QoL.

#### Country

Chilean respondents had poorer outcomes than French family members in physical functioning, general health, physical composite score, mental health, and vitality (Boyer et al., 2012).

#### Social Stigma

The perceived stigma of the caregiver was shown to be associated with a poorer QoL (Chou et al., 2009; Zauszniewski et al., 2009).

#### Day Care

For relatives of patients attending a support service such as day care, in comparison with those not attending, associations were shown with the environment, physical, psychological, and social domains, which are poorer in the relatives of patients attending day care (ZamZam et al., 2011).

#### Social Support

A high level of perceived family support, in terms of instrumental, emotional, and informational support, increases the QoL of relatives (Chou et al., 2009; Leng et al., 2019).

## Discussion

The aim of this study was to identify the current evidence on the factors associated with the QoL of relatives of individuals with SMI. This evidence indicates that there are different factors influencing QoL domains and reflects the complexity of this issue. The findings are in agreement with previous knowledge, as shown below. Given the large number of QoL-related factors that have been found, those that show a higher degree of association will be discussed in this section.

In terms of individual factors, the emerged factors independently associated with the relative’s QoL in the multivariate analyses were: age, educational level, employment status, and physical health.

Older relatives have been shown to have a poorer QoL. It is reasonable to expect that, as a consequence of aging, their own state of health has negatively influenced their independence and physical health, which determines a significantly poorer QoL (Alcañiz & Solé-Auró, 2018). The higher QoL outcomes of employed respondents are also supported by previous studies. This could be explained by the positive financial consequences, the social aspects of their lives, as well as a greater self-esteem and service satisfaction (Gold et al., 2016).

Sense of coherence had previously been shown to be associated with physical and psychological well-being. Relatives with a high sense of coherence were probably focusing on the positive aspects of the difficulties arising in their lives, considering their life to have meaning, and understanding and accepting the SMI, which prevents them from having negative feelings about their caregiving role (Chittem et al., 2015). Personal resourcefulness, which is comprised of positive thinking, problem-solving, self-control, and self-help skills, is related to a difference in QoL. Personal resourcefulness has shown to be an indicator of family resilience, associated with more adaptive functioning (Hine et al., 2018). In addition, individuals with good health usually perceive their QoL to be better. Unhealthy relatives would be less able to perform their caregiving tasks and would thus face more difficulties (Caqueo-Urízar et al., 2014; Ribé et al., 2018).

The family factors that emerged as independently associated with QoL were the degree of kinship, household income, and living with the patient. Regarding the relative’s relationship to the patient, parents often step in to fill the gaps in the healthcare service system. Compared to other family relationships, the higher emotional engagement and attachment between parents and children could motivate worries about possible relapses, feelings of personal responsibility for the illness due to parenting, worries regarding the future related to their permanent responsibility for the patient, and the question of who will take care of the patient if they no longer can (Hasson-Ohayon et al., 2019; Jungbauer et al., 2003).

In turn, the characteristics of the patient with SMI factors that are independently associated with QoL in multivariate analyses were: the patient’s educational level, functioning and psychopathological status, and a diagnosis of schizophrenia. As for the factors that emerged as independently associated with the relative’s QoL in multivariate analyses, these were related to the course of illness, social readjustment, illness perception, the relative’s depression symptoms, and the duration and onset of the illness. Burden has previously been shown to negatively affect QoL.

The relative may experience a burden in providing services to their family member, as well as in relation to the required household chores and other family responsibilities (Caqueo-Urízar et al., 2014; Zeng et al., 2017). Regarding distress, it is expected that relatives of individuals with SMI are themselves vulnerable to mental disorders and distress due to genetic covariance and similarities in their environment (Cross-Disorder Group of the Psychiatric Genomics Consortium, 2013). Finally, the contextual factors independently associated with QoL were: country, perceived social support, perceived stigma, and attending day care.

### Strengths and Limitations

The findings have direct implications for mental health professionals, highlighting the importance of examining the impact of factors associated with the QoL of relatives in order to develop interventions tailored to the characteristics of the patients and their relatives.

Non-modifiable factors, such as age, degree of kinship, or employment status, can be systematically assessed in clinical practice to detect possible associations with poor QoL. The population presenting these factors should be the target of individualized interventions. Health professionals may also focus on modifiable factors in order to provide psychoeducation or support group interventions, which have been shown to be effective in providing family members with a better understanding of the facilitating factors and barriers to their QoL and well-being (Sin et al., 2017; Yesufu-Udechuku et al., 2015).

Scientifically valid and quality results on the QoL of relatives of individuals with SMI are summarized, which can serve as a basis and guide for future research and practice interventions. However, some limitations have been identified in this review, including limitations concerning the studies reviewed. Firstly, a cross sectional design is unable to reveal cause-effect relationships. In addition, the instruments used are generic measures of QoL that were not specifically tailored for relatives of adults with SMI. No meta-analysis was conducted for reasons related to the studies included, such as the disparate factors evaluated or the dissimilarity of outcome measures.

In addition, given the variability of the measures and presentation of results in the included studies, it was not possible to use the same effect estimator or to determine the confidence interval of the effect estimators, which would have enhanced the quality of this systematic review. Finally, the groups of participants from specific areas consisted of volunteers in many studies, which may not be representative of the family population.

For future research, it may be interesting to consider in greater depth the cultural, economic, and social factors that may contribute to differences in QoL, as well as the therapeutic alliance and adherence to therapy. Confounding factors that may be influencing QoL, such as the time spent in care or whether the family received any assistance during the course of illness, should also be taken into account. Longitudinal studies with larger and more representative samples of relatives of individuals with SMI should be conducted to capture changes in QoL over time related to the variation of different factors such as the ones that have been found in this review.

The impact of several factors on the relatives’ QoL is substantial. These include personal and family characteristics, social skills and support, the factors of the individual with a mental illness, care experience, and the therapeutic relationship with health professionals. These factors should be studied in future research in order to gain more in-depth knowledge of the well-being and health of the family members of an individual with SMI.
